# Changes in Chinese Discharged Chronic Mental Patients Attending a Psychiatric Rehabilitation Program with Holistic Care Elements: A Quasi-Experimental Study

**DOI:** 10.1100/tsw.2006.335

**Published:** 2006-02-16

**Authors:** Andrew L. Luk, Daniel T.L. Shek

**Affiliations:** Kiang Wu Nursing College of Macau and Social Welfare Research and Practice Centre, Department of Social Work, The Chinese University of Hong Kong, Hong Kong

**Keywords:** psychiatric rehabilitation, self-help group, holistic care, Chinese discharged chronic mental patients

## Abstract

This study attempted to examine the changes and related factors in discharged chronic mental patients attending a psychiatric rehabilitation program in Hong Kong adopting a self-help group (SHG) approach with holistic care emphases on the physical, psychological, social, and spiritual functioning of the program participants. A quasi-experimental design involving an experimental group (109 participants attending the program) and a control group (154 patients from a psychiatric outpatient clinic who had never attended any SHG before) was adopted with the participants responding to measures assessing their functioning in the physical, psychological, social, and spiritual domains. Results showed that those who joined the SHG with holistic care elements had more friends and more social satisfaction than the control subjects. Duration of attendance, religious involvement, and group involvement were three key factors related to the outcomes of the program participants. This pioneering study provides support for the effective use of the SHG approach with holistic care elements to help discharged chronic mental patients in the Chinese culture.

## INTRODUCTION

It is well documented that most patients with chronic mental illness suffer from severe psychosocial dysfunction[1,2]. The primary dysfunction is directly due to psychiatric symptoms like persistent hallucination and delusion, and the adverse psychological reactions such as low self-esteem and helplessness in response to one's illness. The secondary dysfunction is caused by stigmatization attached to being a mental patient that makes discharged chronic mental patients difficult to integrate into the community[3]. Besides psychosocial dysfunction, some local and overseas studies suggest that many mental patients have spiritual problems, such as a lack of hope and loss of meaning and purpose of life[2,4,5].

As medicine moves forward in the 21$^{st}$ century, a new conception of the nature of human beings is emerging in which the biopsychosocial domain is enlarged by the addition of the spiritual dimension, which redefines the nature and purpose of treatment and care to patients. In the nursing literature, nursing colleagues suggest that acute and chronic illnesses frequently cause disharmony of the body, mind, and spirit, such as a loss of identity, self-control, acceptance, and meaning of life[6,7]. In the traditional biopsychosocial perspective, a patient's behavior is seen as influenced by both personal (biological and psychological factors) and social factors. Nevertheless, with the emergence of views that emphasize the spiritual dimension (e.g., transpersonal psychology) and the fact that religion plays an important role for doctors and patients, there are views suggesting that one should include the spiritual dimension in order to understand human nature. In the social work literature, there has been an increasing emphasis on the importance of addressing the spiritual needs of the clients and the principle that the spiritual dimension should be taken into consideration in social work intervention is gradually upheld by social work practitioners[8,9,10,11]. In the counseling literature, there are also new counseling models in which the spiritual dimension is included[12]. As pointed out by Anderson and Young[13], "all clinicians inevitably face the challenge of treating patients with religious troubles and preoccupation" (p. 532). Aldridge[14] also argued that many clinical cases with the issues of abandonment, suffering, loss of hope and meaning, and the transitions from living to dying are essentially spiritual, and clearly not solely physiological, psychological, or social in nature. In a national survey by Bergin and Jensen[15] with samples including psychologists, psychiatrists, social workers, and marriage and family therapists, 29% agreed that religious issues were important in the treatment of all or many of their clients. In the U.S., "religious or spiritual problem" has been established as a psychiatric diagnosis[16] while "spiritual distress" is also approved as a nursing diagnosis[17].

Although there are different views on the concept of spirituality and different ingredients have been proposed to define the concept, sense of hope and purpose in life are commonly regarded as important components of spirituality. Highfield and Cason[18] proposed that there are four types of spiritual needs: need for meaning and purpose in life, need to receive love, need to give love, and need for hope and creativity. Thompson[19] outlined five types of spiritual distress: spiritual pain, spiritual alienation, spiritual guilt, spiritual loss, and spiritual despair (loss of hope). In their proposed framework on spiritual assessment, Anandarajah and Hight[20] suggested that hope and life meaning and their sources should be assessed. In short, the sense of hope and its loss (i.e., hopelessness), as well as purpose in life, are important spiritual elements to be considered by psychiatric rehabilitation professionals.

Despite the growing emphasis of holistic health care in mainstream medicine[21], the application of holistic health care principles and elements in psychiatric rehabilitation has been limited. Although spiritual element is included in some popular intervention models such as that for Alcoholics Anonymous (AA), the effectiveness of such models are seldom systematically examined. For example, with specific reference to social work, a search of the publications indexed by the Social Work Abstracts up to May 2005 showed the following results: (1) using the search terms of "religion" and "psychiatric", there were only seven citations; (2) using the search terms of "spiritual" and "psychiatric", there were only eight citations; (3) using the search terms of "holistic" and "psychiatric", there were only eight citations. The results based on these searches clearly show that research studies examining psychiatric rehabilitation based on a holistic approach are grossly inadequate in the social work literature.

With specific reference to Hong Kong, an examination of psychiatric rehabilitation in Hong Kong shows several characteristics[22,23,24]. First, the rehabilitation model adopted is basically medically oriented, with chemotherapy as the major emphasis. Second, it lacks structured rehabilitation programs, especially informal community programs based on the "self-help group" approach (SHG). Third, with the exception of the gospel drug rehabilitation programs[25], spiritual element is not consciously included in most of the psychiatric rehabilitation programs. Fourth, with very few exceptions[25], there are few studies documenting the changes of participants attending psychiatric rehabilitation programs adopting self-help and holistic care principles.

There are some isolated services in Hong Kong that attempt to provide holistic psychiatric rehabilitation service to mental patients. One of the service providers is Christian Oi Hip Fellowship (Fellowship) that is a Christian non-government organization providing holistic care programs for discharged chronic mental patients. The program is characterized by a SHG approach and holistic care in which the physical, psychological, social, and spiritual needs of the clients are emphasized, where holistic care is conceived as the care delivered to meet an integral, independent individual's health needs including physical, psychosocial, spiritual, cultural, and environmental aspects as a whole[26]. Beyond using solely conventional therapeutic intervention such as psychodynamic, cognitive, and behavioral strategies in running SHGs, intervention addressing the spiritual needs of the participants in the form of hymn singing, Bible reading, personal sharing, and intercessions are also used in the Fellowship (i.e., focus on spiritual changes beyond changes in the biopsychosocial dimension). This holistic care rehabilitation program in the Fellowship seeks to integrate Christian beliefs into every aspect of one's life through regularly scheduled meetings for mutual support. All volunteer group counselors, mostly professionals (including psychiatrists and psychiatric nurses), but some paraprofessionals, are Christians who take care of their group members as a whole person including body, mind, and spirit. Established in 1990, the Fellowship has formed 12 SHGs with more than 120 members actively participating in these groups. The first author has been the chairman of the Fellowship for the past years. Although the Fellowship focuses on the spiritual dimension of a person, understanding a patient's responses in terms of demon possession or evil spirit is not the basic focus of the Fellowship.

Regarding the format of the Fellowship, a group meeting is held once every fortnight. One meeting is for open sharing and praying. Another meeting is used for sharing a special topic of concern by all members. In the design of a new group in the Fellowship, the development of the group is roughly divided into four stages. In the first 6 months of the initial stage, a new member has to get familiar with the other members and the group norm. A manual of stress management developed by the Fellowship will be used. The second stage is development stage from 6 months to 2 years, when one develops trust in others, determines if he or she can mix well in the group, and becomes more involved in the group activities. The third stage is the mature stage from 2–5 years when members choose to stay behind and will usually be invited to become the committee members of their respective groups, where they will learn to plan the group meetings and to care for the newcomers. During this period, some groups can be independent and maintain their own activities without much involvement of the group counselors. The final stage is the self-sustaining stage when the group has been running for more than 5 years and develops its own programs or activities. Some key members of the group would maintain the norm of their respective groups and take an active role in caring for the newcomers.

With the strong tradition of "medical model" in Hong Kong that emphasizes the treatment of the body in terms of physical symptoms together with the missing dimension on the spiritual care, the holistic approach adopted by the Fellowship in providing a holistic care rehabilitation program is worthy of investigation. In addition, as Chinese people constitute roughly one-fifth of the world's population, it would be important to examine systematically how psychiatric rehabilitation with the inclusion of the spiritual dimension may help the discharged chronic mental patients of China.

The fundamental question posed in this study is concerned with the changes in the participants attending the Oi Hip Fellowship that adopts a holistic-type health care in psychiatric rehabilitation. Another research question addressed in this study is what factors are related to the outcomes of participants attending the Oi Hip Fellowship. A review of the literature shows that some factors — such as marital status, employment, duration of attendance in the group, religious conversion, religious and group involvement — are related to the outcomes of clients in psychiatric rehabilitation. In this study, several factors that are more closely related to holistic care programs including the SHG and religious elements are examined. These include: (1) duration of attendance (including stages) in the group, (2) religious involvement, and (3) group involvement.

Concerning duration of attendance, Powell[27] showed that the primary purpose of the SHG was for long-term mutual support and people would benefit after the acute condition. Regarding religious involvement, Pargament and Brant[28] summarized the results of 46 studies that examined the relationship between religious orientations and the outcomes to critical life events. It was found that high levels of church involvement, personal religious beliefs, faith, and a more intrinsic religious commitment were related to beneficial outcomes. For group involvement, Norton et al.[29] examined the perceived costs and benefits of membership in a SHG and they found that active members reported significantly higher benefits than less active members. Based on the above-mentioned literature, it would be expected that: (1) members who have stayed for a longer duration (i.e., later stages of the program) would have better outcomes than those who have stayed for a shorter duration (i.e., earlier stages of the program), (2) those who had more religious involvement would have better outcomes than those with less religious involvement, (3) those who have more group involvement would have better functioning compared with those with less group involvement.

## METHODS

A quasi-experimental design with an experimental group and a matched control group was adopted in this study to examine the changes in the participants joining the Oi Hip Fellowship. For the experimental group, 109 members who had attended the SHGs in the Fellowship during the recent month at the time of data collection were recruited (n = 109). One of the group meetings was used for members to complete the self-administered questionnaire with a brief explanation from the respective group counselors. Regarding the control group (n = 154), written approval was obtained from the hospital management to recruit discharged chronic mental patients from a psychiatric outpatient clinic in a regional general hospital. Potential respondents were first matched with the background of the experimental group members as far as possible (including diagnosis, age, sex, duration of illness, and marital status). When the potential participants attended the clinic, they were invited to participate in the study by filling in the same self-administered questionnaire completed by the experimental subjects. Voluntary participation and confidentiality were assured in both groups. The outcome measures of these two groups were then compared.

### Instruments

There are seven sections in the self-administered questionnaire. Section 1 to Section 6 contained assessment tools with demonstrated validity and reliability measuring the outcomes of the participants, including their functioning in the physical, psychological, social, and spiritual domains. The last section contained self-devised questions assessing the demographic background of the participants. The outcome measures are as follows:

1. **General Health Questionnaire (GHQ).** The GHQ was developed to measure psychiatric symptoms[30]. The Chinese GHQ was found to compare favorably with the English version at the scale level[31] and evidence showed that the GHQ possesses acceptable psychometric properties[32,33,34]. A higher GHQ scale score indicates a higher level of psychiatric symptoms.
2. **Rosenberg Self-Esteem Scale (ESTEEM).** The ESTEEM consists of ten items and has been used to measure self-worth[35]. The Chinese Rosenberg Self-Esteem Scale has been developed by Shek with an acceptable reliability[36]. A higher ESTEEM scale score indicates a higher level of self-esteem.
3. **Social Support Questionnaire-6 (SSQ-6).** The SSQ-6 was the short form developed by Sarason et al.[37] by selecting 6 items most representative of the SSQ-27 items. It was designed to quantify the number of support persons available (SSQ-Number) as well as the corresponding perceived satisfaction (SSQ-Satisfaction). The SSQ-6 was found to have high internal reliability for both SSQ-Number and SSQ-Satisfaction (0.90–0.93). The SSQ-6 was later translated into Chinese[38] and adequate reliability of the scale was found. A higher SSQ-Number score indicates more number of support persons available and a higher level of SSQ-Satisfaction indicates a higher level of perceived social satisfaction.
4. **Purpose in Life Questionnaire (PIL).** The PIL was designed to quantify existential meaning perceived by an individual[39]. The Chinese version of the PIL was translated by Shek[40] and high reliability and validity of the Chinese PIL have been reported[41]. A higher PIL scale score indicates a higher level of perceived life meaning.
5. **Hopelessness Scale (HOPE).** The HOPE was a 20-item scale developed by Beck et al.[42] to quantify the degree of pessimism in an individual. The reliability and validity of the scale have been demonstrated by Beck et al.[42]. The Chinese version of the Hope (C-Hope) was developed by Shek[43] who found that the scale possessed acceptable psychometric properties. A higher HOPE scale score indicates a higher level of hopelessness in this study.
6. **Hong Kong Chinese Version of the World Health Organization Quality of Life Measure (WHOQOL-BREF[HK]).** WHOQOL-BREF(HK) is the shortened form with 28 items of the WHOQOL-100 designed as a universal tool for measuring quality of life in clinical setting and health service program evaluations. One question is used to measure the overall QOL (QOL-OVERALL) and another one for overall health. The rest of the questions are divided into four domains to measure QOL in the physical (QOLDOM1), psychological (QOLDOM2), social (QOLDOM3), and environmental (QOLDOM4) aspects, with 7, 8, 3, and 8 questions, respectively. The internal consistency, test-retest reliability, concurrent validity, and construct validity of the WHOQOL-BREF(HK) have been well established[44].

With reference to the above measures, the categorization of outcome indicators for the present study is as follows: physical well being (indexed by QOLDOM1), psychological well being (indexed by the GHQ, ESTEEM, and QOLDOM2), social well being (indexed by SSQ-Number, SSQ-Satisfaction, and QOLDOM3), and spiritual well being (indexed by PIL and HOPE). It was expected that if the Fellowship were beneficial to the program participants, the outcome indicators in the experimental group participants would have better physical, psychological, social, and spiritual functioning than did the control group participants.

## RESULTS

Analyses showed that while the two groups did not differ in gender ratio, age, living status, and last-year hospital admission, significant differences between the two groups were found in the areas of marital status, education, job status, religion, diagnosis, year of illness, total previous hospital admission, and medication requirement. The details of the demographic characteristics of the two groups can be seen in Table 1.

Because the two groups differed on several background demographic and health-related variables, analyses of covariance analyses were carried out to examine the differences between the two groups in terms of the outcome indicators by removing the effects of the covariates, including marital status, education, job status, religion, diagnosis, year of illness, total previous hospital admission, and medication requirement. This procedure could reduce the confounding effects of the demographic variables on the outcome measures.

Results showed that in the social domain, the experimental group displayed higher mean scores in SSQ-Number ($F[245] = 11.73$, $p < 0.001$) and SSQ-Satisfaction ($F[222] = 3.75$, $p = 0.05$) than did the control group. Furthermore, the experimental group scored higher in the environmental domain of the Quality of Life Scale (QOLDOM4) than did the control group ($F[237] = 5.338$, $p < 0.05$). Although there were no statistically significant differences between these two groups in terms of the remaining indicators, the findings were in the predicted direction. Analysis using the binominal test showed that based on the 11 outcome measures ($n = 11$), all the means of the experimental group were better than those of the control group in the predicted direction ($p < 0.01$).

Regarding the relationship between duration of attendance and the outcome indicators, results showed that those who stayed longer in the program only displayed higher levels of self-esteem ($r = 0.29$, $p < 0.005$). In addition, Table 2 shows that those who were at the later stages of the program had higher ESTEEM and SSQ-Satisfaction scores than did those who were at the earlier stages of the program.

Although there were no differences in the program participants in different stages of the program in terms of the GHQ, PIL, and HOPE scores, the findings showed that there was a gradual trend in the improvement of the psychological and spiritual functioning of the participants.

The findings presented in Table 3 show that high levels of religious involvement (indexed by higher frequencies of attendance) were generally related to better outcomes in the physical, psychological, social, and spiritual domains (except SSQ-Number and QOLDOM3). However, because poor mental health in terms of the presence of psychiatric symptoms might affect a member’s attendance in church activities, partial correlation analyses were conducted to test the relationship between church attendance and changes in functioning with GHQ as a covariate to control the effect of mental health. Results showed that there were significant positive correlations between church attendance and QOLDOM1 ($r = 0.197$, $p = 0.05$), ESTEEM ($r = 0.238$, $p < 0.05$), and QOL-OVERALL ($r = 0.206$, $p < 0.05$), and a significant negative correlation between the church attendance and HOPE ($r = –0.221$, $p < 0.05$) was found. Details of the result are presented in Table 3.

**TABLE1**
**Demographic Data of the Treatment Group and the Control Group**

| Variables | | Treatment Group (n = 109) | Control Group (n = 154) | Chi-Square |
|---|---|---|---|---|
| Sex | Male: | 55(50.5%) | 82(53.9%) | NS |
| | Female: | 54(49.5%) | 70(46.1%) | |
| Age | Below 25 | 4(4.0%) | 9(6.3%) | NS |
| | 25–34 | 33(32.6%) | 32(22.4%) | |
| | 35–44 | 43(42.6%) | 68(47.5%) | |
| | 45–54 | 21(20.8%) | 32(22.4%) | |
| | 55 and above | Nil | 2(1.4%) | |
| Marital status | Single | 74(68.5%) | 70(45.8%) | ** |
| | Married | 25(23.1%) | 65(42.5%) | |
| | Others | 9(8.3%) | 18(11.8%) | |
| Education | Primary or below | 5(4.6%) | 43(27.9%) | *** |
| | Secondary | 89(81.7%) | 98(63.6%) | |
| | University and postgraduate | 15(13.7%) | 13(8.4%) | |
| Job status | Waiting for employment | 38(35.2%) | 46(30.1%) | ** |
| | Full time | 32(29.6%) | 33(21.6%) | |
| | Part time | 16(14.8%) | 9(5.9%) | |
| | Others | 22(20.4%) | 65(42.5%) | |
| Living status | Alone | 11(10.1%) | 21(14.1%) | NS |
| | With family members | 90(82.6%) | 120(80.5%) | |
| | Hostel | 6(5.5%) | 7(4.7%) | |
| | Others | 2(1.8%) | 1(0.7%) | |
| Religion | Nil | 5(4.6%) | 87(56.5%) | *** |
| | Christian | 102(93.6%) | 33(21.4%) | |
| | Non-Christian | 2(1.8%) | 34(22.1%) | |
| Diagnosis | Schizophrenia | 72(66.1%) | 64(43.0%) | *** |
| | Manic depressive | 24(22.0%) | 12(8.1%) | ** |
| | Depression | 18(16.5%) | 48(32.2%) | * |
| | Others | 16 | 46 | NS |
| Year of illness | 1–5 years | 17(15.6%) | 61(39.6%) | *** |
| | 6–10 years | 17(15.6%) | 29(18.8%) | |
| | 11–15 years | 22(20.2%) | 16(10.4%) | |
| | 16–20 years | 17(15.6%) | 16(10.4%) | |
| | 21–25 years | 11(10.1%) | 2(1.3%) | |
| | 26 and above | 25(22.9%) | 30(19.5%) | |
| Total previous hospital admission | Nil | 21(19.6%) | 58(38.9%) | * |
| | Once | 22(20.6%) | 30(20.1%) | |
| | Twice | 21(19.6%) | 21(14.1%) | |
| | Three and four times | 23(21.5%) | 23(15.5%) | |
| | Five times and above | 20(18.7%) | 17(11.4%) | |
| Last-year hospital admission | Nil | 83(79.0%) | 105(70.9%) | NS |
| | Once or above | 22(21%) | 43(29.1%) | |
| On medication | Required | 108(99.1%) | 141(93.4%) | * |
| | Not required | 1(0.9%) | 10(6.6%) | |

NS: not statistically significant, * $p < 0.05$, ** $p < 0.005$, *** $p < 0.001$.

**TABLE 2**
**Differences in Functioning among Participants at Different Stages**

| Measures | Stages | n | Mean Score | SD | F | Sig. |
|---|---|---|---|---|---|---|
| QOLDOM1 | Below 6 months | 10 | 11.60 | 1.86 | 1.405 | 0.245 |
| | 6 months to 2 years | 23 | 12.81 | 2.00 | | |
| | 2–5 years | 38 | 13.31 | 2.63 | | |
| | Over 5 years | 38 | 12.96 | 2.36 | | |
| GHQ | Below 6 months | 10 | 35.70 | 12.15 | 1.108 | 0.349 |
| | 6 months to 2 years | 23 | 30.91 | 14.18 | | |
| | 2–5 years | 38 | 29.86 | 16.83 | | |
| | Over 5 years | 38 | 27.00 | 11.58 | | |
| ESTEEM | Below 6 months | 10 | 24.00 | 3.91 | 2.664 | 0.05 |
| | 6 months to 2 years | 23 | 25.21 | 4.61 | | |
| | 2–5 years | 38 | 26.47 | 4.77 | | |
| | Over 5 years | 38 | 27.89 | 4.76 | | |
| QOLDOMH2 | Below 6 months | 10 | 10.65 | 2.68 | 1.809 | 0.15 |
| | 6 months to 2 years | 23 | 12.36 | 3.29 | | |
| | 2–5 years | 38 | 12.82 | 2.70 | | |
| | Over 5 years | 38 | 12.63 | 2.24 | | |
| SSQ-Number | Below 6 months | 10 | 1.55 | 0.94 | 0.543 | 0.654 |
| | 6 months to 2 years | 23 | 2.09 | 1.45 | | |
| | 2–5 years | 37 | 2.29 | 1.70 | | |
| | Over 5 years | 37 | 2.16 | 1.82 | | |
| SSQ-Satisfaction | Below 6 months | 9 | 4.01 | 1.04 | 3.558 | 0.017 |
| | 6 months to 2 years | 23 | 4.86 | 0.79 | | |
| | 2–5 years | 35 | 4.59 | 0.73 | | |
| | Over 5 years | 36 | 4.87 | 0.70 | | |
| QOLDOM3 | Below 6 months | 10 | 11.06 | 1.78 | 1.517 | 0.214 |
| | 6 months to 2 years | 23 | 12.36 | 3.52 | | |
| | 2–5 years | 38 | 12.52 | 1.94 | | |
| | Over 5 years | 38 | 12.94 | 2.45 | | |
| PIL | Below 6 months | 10 | 75.20 | 21.70 | 1.676 | 0.177 |
| | 6 months to 2 years | 23 | 86.65 | 28.33 | | |
| | 2–5 years | 38 | 91.76 | 23.18 | | |
| | Over 5 years | 38 | 92.52 | 20.88 | | |
| HOPE | Below 6 months | 10 | 62.50 | 11.23 | 0.773 | 0.512 |
| | 6 months to 2 years | 23 | 62.95 | 17.52 | | |
| | 2–5 years | 37 | 57.37 | 12.78 | | |
| | Over 5 years | 38 | 58.47 | 17.87 | | |
| QOL-OVERALL | Below 6 months | 10 | 3.30 | 1.34 | 0.178 | 0.911 |
| | 6 months to 2 years | 23 | 3.13 | 1.06 | | |
| | 2–5 years | 38 | 3.29 | 0.87 | | |
| | Over 5 years | 38 | 3.18 | 0.77 | | |

*Note:* QOLDOM1: Quality of Life Domain 1; GHQ: General Health Questionnaire; ESTEEM: Rosenberg Self-Esteem Scale; QOLDOMH2: Quality of Life Domain 2 Hong Kong; SSQ-Number: Social Support Questionnaire-Number; SSQ-Satisfaction: Social Support Questionnaire-Satisfaction; QOLDOM3: Quality of Life Domain 3; PIL: Purpose in Life Questionnaire; HOPE: Hopelessness Scale; QOL-OVERALL: Quality of Life-Overall.

**TABLE 3**
**Religious Involvement (Church Attendance per Month) and Outcomes Based on Pearson and Partial Correlation Analyses**

| | | Pearson's r | | | Partial Correlation | | |
|---|---|---|---|---|---|---|---|
| **Scales** | **n** | **r** | **Sig. (2-Tailed)** | **Effect Size** | **R** | **Sig. (2-Tailed)** | **Effect Size** |
| QOLDOM1 | 98 | 0.270 | 0.007** | 0.07 | 0.197 | 0.053 | 0.03 |
| GHQ | 99 | –0.211 | 0.036* | 0.04 | | (covariate) | |
| ESTEEM | 99 | 0.313 | 0.002** | 0.09 | 0.238 | 0.018* | 0.05 |
| QOLDOMH2 | 98 | 0.256 | 0.011* | 0.06 | 0.167 | 0.102 | 0.02 |
| SSQ-Number | 97 | 0.095 | 0.354 | 0.01 | 0.062 | 0.550 | 0.00 |
| SSQ-Satisfaction | 94 | 0.249 | 0.015* | 0.06 | 0.195 | 0.061 | 0.03 |
| QOLDOM3 | 98 | 0.167 | 0.101 | 0.02 | 0.103 | 0.317 | 0.01 |
| PIL | 99 | 0.267 | 0.008** | 0.07 | 0.180 | 0.076 | 0.03 |
| HOPE | 98 | –0.288 | 0.004** | 0.08 | –0.221 | 0.029* | 0.04 |
| QOL-OVERALL | 99 | 0.263 | 0.008** | 0.07 | 0.206 | 0.042* | 0.04 |

*Note:* QOLDOM1: Quality of Life Domain 1; GHQ: General Health Questionnaire; ESTEEM: Rosenberg Self-Esteem Scale; QOLDOMH2: Quality of Life Domain 2 Hong Kong; SSQ-Number: Social Support Questionnaire-Number; SSQ-Satisfaction: Social Support Questionnaire-Satisfaction; QOLDOM3: Quality of Life Domain 3; PIL: Purpose in Life Questionnaire; HOPE: Hopelessness Scale; QOL-OVERALL: Quality of Life-Overall.

* $p < 0.05$; ** $p < 0.01$.

Finally, as revealed in Table 4, the present findings showed that there were significant differences between the committee members and the noncommittee members in terms of the outcome indicators (except QOLDOM4 and QOL-OVERALL).

## DISCUSSION

Adopting a quasi-experimental design, this study examined the physical, psychological, social, and spiritual functioning of discharged chronic mental patients attending a psychiatric rehabilitation program adopting holistic care elements (experimental group) in comparison with those in the control group. Results showed that the SSQ-Number and SSQ-Satisfaction scores in the experimental group were higher than those of the control group. This observation is generally consistent with the previous research findings that SHG could benefit the social functioning of the group members. In the study by Trojan[45], most of the members reported considerable positive changes in personal network, including relationship with family and friends. The study by Humphreys and Noke[46] showed that participation in mutual-help group could help to increase the number of close friends. The present findings are consistent with the qualitative findings reported by Luk[47] that a majority of the informants regarded the Fellowship as a group for long-term and effective mutual support and that they could acquire mutual support and increased number of friends from the Fellowship. Because very few studies have examined the social benefits of joining psychiatric rehabilitation programs adopting SHG approach with holistic healthcare elements, the present findings can be regarded as pioneering, particularly in the Chinese culture.

**TABLE 4**
**Differences Between Committee Members and Noncommittee Members in Terms of the Outcome Indicators**

| Scales | Committee Member | n | Mean Score | SD | t | Sig. (2-Tailed) | Effect Size |
|---|---|---|---|---|---|---|---|
| QOLDOM1 | Yes<br>No | 63<br>44 | 13.34<br>12.38 | 2.41<br>2.20 | 2.08 | 0.04* | 0.047 |
| GHQ | Yes<br>No | 63<br>45 | 26.80<br>33.44 | 12.41<br>15.90 | –2.43 | 0.01* | 0.056 |
| ESTEEM | Yes<br>No | 63<br>45 | 27.77<br>24.68 | 4.83<br>4.15 | 3.46 | 0.001** | 0.107 |
| QOLDOMH2 | Yes<br>No | 63<br>44 | 13.03<br>11.65 | 2.14<br>2.96 | 2.65 | 0.009** | 0.063 |
| SSQ-Number | Yes<br>No | 61<br>45 | 2.42<br>1.79 | 1.79<br>1.31 | 1.99 | 0.048* | 0.038 |
| SSQ-Satisfaction | Yes<br>No | 59<br>44 | 4.83<br>4.52 | 0.67<br>0.91 | 2.00 | 0.047* | 0.045 |
| QOLDOM3 | Yes<br>No | 63<br>44 | 12.93<br>11.93 | 2.12<br>2.94 | 2.02 | 0.045* | 0.038 |
| PIL | Yes<br>No | 63<br>45 | 94.34<br>82.80 | 23.55<br>22.69 | 2.55 | 0.012* | 0.061 |
| HOPE | Yes<br>No | 62<br>45 | 56.14<br>63.64 | 16.37<br>13.62 | –2.50 | 0.014* | 0.059 |
| QOLDOM4 | Yes<br>No | 63<br>43 | 12.92<br>12.33 | 2.11<br>2.57 | 1.27 | 0.20ns | 0.015 |
| QOL-OVERALL | Yes<br>No | 63<br>45 | 3.30<br>3.11 | 0.84<br>1.07 | 1.03 | 0.19ns | 0.023 |

*Note:* QOLDOM1: Quality of Life Domain 1; GHQ: General Health Questionnaire; ESTEEM: Rosenberg Self-Esteem Scale; QOLDOMH2: Quality of Life Domain 2 Hong Kong; SSQ-Number: Social Support Questionnaire-Number; SSQ-Satisfaction: Social Support Questionnaire-Satisfaction; QOLDOM3: Quality of Life Domain 3; PIL: Purpose in Life Questionnaire; HOPE: Hopelessness Scale; QOL-OVERALL: Quality of Life-Overall.

* $p < 0.05$; ** $p < 0.01$; ns: nonsignificant.

Contrary to our expectation, the two groups did not differ in terms of the physical, psychological, and spiritual outcome indicators. There are several factors contributing to this observation. First, as stigmatization attached to discharged chronic mental patients would make them difficult to integrate into the community[3], overcoming social isolation might be the most difficult task faced by the program participants (e.g., [48,49]). As a result, changes in the social domain alone could be regarded as a more prominent area of achievement as compared to other areas. Second, as many discharged chronic mental patients could still maintain a satisfactory physical life such as eating and sleeping well, and without any physical problems, this may explain the lack of significant differences between the experimental group and control group in the physical domain. Finally, because no baseline data had been collected, the adoption of the nonequivalent group design may make the comparison between the two groups insensitive. Nevertheless, it is noteworthy that although the two groups did not differ significantly on the outcome indicators in the physical, psychological, and spiritual domains, the differences between the two groups were in the predicted direction. These findings are basically positive and they strongly suggest that a more rigorous design (e.g., addition of pretest measures) should be used in future studies.

Although self-esteem was the only indicator that was related to duration of joining the groups, that is a unique finding because no systematic study has been conducted to examine this factor in long-term SHG for the severe mentally ill both in the Chinese and Western contexts. Regarding the changes of functioning among different stages in the group, only the ESTEEM and SSQ-Satisfaction scores showed statistically significant differences in the different stages of the program (Table 2). One possible factor that contributes to this observation is that the demarcation of the different stages of the group is roughly divided. Perhaps more research should be conducted to examine this issue further.

Religious involvement was also found to be a factor related to the functioning of the program participants. Although the use of frequency of church attendance as an indicator of religious involvement may be quite rough, it can still be regarded as a good reflection of how involved a member was. Results showed that the frequency of church attendance (i.e., religious involvement) was significantly related to nearly all aspects of functioning. The relationship between religious involvement and psychological well being was still observed even when the effect of psychiatric morbidity was removed. The present findings are generally consistent with previous research findings that religious involvement is positively related to psychological well being[28,50,51]. In a recent meta-analytic study, Powell, Shahabi, and Thoresen[52] also concluded that there were research findings supporting the linkage between religion and spirituality and physical health.

With regard to group involvement and the outcome indicators, the present finding showed that there were differences between the active and the less active members in their physical, psychological, social, and spiritual domains. The present findings are consistent with the previous research findings[29,53]. Because mutual support is the essence of all SHGs and the design of the SHG emphasizes the involvement of every member of the group, it is why shared leadership and an emphasis on empowerment have been found to be the characteristics of a successful SHG[53]. In a study of the perceived costs and benefits of membership in a SHG, Norton et al.[29] showed that more active members reported significantly higher benefits than did the less active members. This may explain why more active involvement was associated with better adjustment.

There are several implications of the present findings for psychiatric rehabilitation work. First, the present findings demonstrate the importance to examine empirically the utility of a holistic care model in psychiatric rehabilitation. In contrast to some of the holistic models in psychiatric rehabilitation (e.g., Alcoholics Anonymous) in which systematic evaluation is seldom carried out, the present study underscores the importance of evaluating the utility of applying principles of holistic care in the psychiatric context. In response to the small number of published studies in this area, particularly in different Chinese contexts, the present study can be regarded as an interesting addition to the literature.

Second, the present findings suggest that there is a need to examine further how religious resource and the spiritual dimension can be integrated into the rehabilitation process of discharged chronic mental patients. In particular, it is important to reinforce the protective role of religion to those clients with religious beliefs. There are studies suggesting that religion is an important resource for clients and their families and that religion was helpful in coping. Pargament and Park[54] found that religion may provide comfort, stimulate personal growth, enhance a sense of intimacy with God, facilitate closeness with others, or offer meaning and purpose in life. Pargament[55] reported six types of religious coping methods (including spiritual coping, congregational coping, religious reframing, religious approaches to agency and control, religious rituals, and combinations of different types) and it was found that 53% of the relationships between religious forms of coping and adjustment were statistically significant. The study by Sullivan[56] also indicated 48% of the successfully rehabilitated, discharged chronic mental patients reported that their spiritual beliefs helped them to solve problems, served as a means of social support, and helped them to make sense out of difficult life dilemmas. In conjunction with these studies, the present study suggests that social work practitioners should further explore how religious resources may help the rehabilitation of discharged chronic mental patients.

Third, the present findings generate some information on the correlates of the functioning of participants in the program. An examination of the literature shows that while there was some discussion on the factors related to the adjustment of the members joining SHGs, there are not much data in the context of psychiatric rehabilitation[45,46,57,58]. As such, the present findings generate research findings that can be used as the bases for formulating conceptual models on the determinants of functioning among discharged chronic mental patients joining programs with holistic care elements. For example, in conjunction with qualitative findings and findings based on the repertory technique, Luk[47] formulated a model on the factors related to the adjustment of discharged chronic mental patients joining psychiatric rehabilitation programs adopting holistic care principles.

Finally, based on the findings on the correlates of the well being of the program participants, the present study has some practical implications on the design of SHGs with holistic care elements for discharged chronic mental patients. First, because the findings show that those who stayed longer had higher self-esteem and those who were at the relatively later stages had better social functioning, how to sustain the motivation of the participants so that they can enjoy the benefits of "prolonged engagement" is an important consideration. Second, because religious involvement was related to well being, it would be practically important to consider how the religious involvement of the clients could be sustained. Finally, because group involvement was related to well being, it is suggested that every newcomer of the group must be briefed about their responsibilities and privileges for the full utilization of the group and the related programs must be well designed so that every member has opportunities to participate actively in the group.

There are several limitations of the study. First, because a quasi-experimental design was used in this study, the differences (or lack of differences) between the experimental group and control group may be due to the intrinsic differences between these two groups that were not assessed in this study. However, because random assignment of participants to the experimental group and control group would not be easy and quasi-experimental design has been commonly used in this context, the adoption of the present design could be regarded as acceptable. The use of ANCOVAs could also help to minimize the confounding effect of the background demographic factors. Second, the relatively better performance of the experimental group may be confounded by sample attrition where members who performed poorly may have dropped out from the group. Fortunately, because only a small number of members dropped out from the Fellowship each year, this confounding effect should not be strong. Third, because a cross-sectional design was used to examine the correlates of the well being of participants who joined Oi Hip Fellowship, the cause-effect relationship between the correlates (e.g., group involvement) and well being is not clear. Definitely, a longitudinal design would yield more meaningful data to illuminate the problem area. In addition, it would be helpful if the effect of the Fellowship could be compared with other intervention (e.g., regular psychosocial intervention with limited number of sessions). Finally, although the present findings show some positive effect of joining the Oi Hip Fellowship, we are not certain why such positive effects took place. Obviously, there is a need to uncover the mechanisms that are conducive to the positive effects observed in future. Despite these limitations, the present findings can be regarded as pioneering in the Chinese psychiatric rehabilitation context.

## ACKNOWLEDGMENTS

This paper is based on the PhD thesis of the first author under the supervision of the second author (Luk, A.L. [2004] The Changes of Ex-Mental Patients Attending a Psychiatric Rehabilitation Program with Holistic Care [Unpublished PhD dissertation]. The Chinese University of Hong Kong.). The preparation of this paper is financially supported by Wofoo Foundation Limited.

## References

1. Hazel, K.L., Herman, S.E., and Mowbray, C.T. (1991) Characteristics of seriously mentally ill adults in a public mental health system. *Hosp. Community Psychiatry* **42**, 518.
2. Lee, P.W.H., Lieh-Mak, F., Yu, K.K., and Spinks, J.A. (1993) Coping strategies of schizophrenic patients and their relationship to outcome. *Br. J. Psychiatry* **163**, 177–182.
3. Wing, J.K. and Morris, B.M. (1981) *Handbook of Psychiatric Rehabilitation Practice*. Oxford University Press, Oxford.
4. Landeen, J., Pawlick, J., Woodside, H., Kirkpatrick, H., and Byrne, C. (2000) Hope, quality of life, and symptom severity in individuals with Schizophrenia. *Psychiatr. Rehabil. J.* **23(4)**, 364–369.

5. Leung, M.H. (1994) The Relation Between Social Support and Social Aadjustment of Schizophrenia Patients in Rehabilitation [Unpublished M.S.W. dissertation]. The Chinese University of Hong Kong.
6. Collitan, M. (1981) The spiritual dimensions of nursing. In *Clinical Nursing: Pathophysiological and Psychosocial Approach*. Beland, I. and Posses, J., Eds. Macmillan, New York.
7. Narayanasamy, A. (1999) A review of spirituality as applied to nursing. *Int. J. Nurs. Stud.* **36**, 117–125.
8. Canda, E.R. (1988) Conceptualizing spirituality for social work: insights from diverse perspectives. *Soc. Thought* **14(1)**, 30–46.
9. Canda, E.R. (1988) Spirituality, religious diversity, and social work practice. *Soc. Casework* **69**, 238–247.
10. Joseph, M. (1987) The religious and spiritual aspects of clinical practice: a neglected dimension. *Soc. Thought* **13(1)**, 12–23.
11. Joseph, M. (1988) Religion and social work practice. *Soc. Casework* **60**, 443–452.
12. Myers, J.E., Sweeney T.J., and Witmer, J.M. (2000) The wheel of wellness counseling for wellness: a holistic model for treatment planning. *J. Couns. Dev.* **78**, 251–266.
13. Anderson, R.G. and Young, J.L. (1988) The religious component of acute hospital treatment. *Hosp. Community Psychiatry* **39(5)**, 528–555.
14. Aldridge, D. (1993) Is there evidence for spiritual healing? ADVANCES, *J. Mind-Body Health* **9(4)**, 4–21.
15. Bergin, A.E. and Jensen, J.P. (1990) Religiosity of psychotherapists: a national survey. *Psychotherapy* **27**, 3–7.
16. American Psychiatric Association (APA) (1994) Diagnostic and Statistical Manual of Mental Disorder. 4th ed. Washington, D.C.
17. Kim, M.J., McFarland, G.K., and McLane, A.M. (1987) *Pocket Guide to Nursing Diagnosis*. CV Mosby, St. Louis.
18. Highfield, M.F. and Cason, C. (1983) Spiritual needs of patients: are they recognized? *Cancer Nurs*. **6**, 187–192.
19. Thompson, I. (2002) Mental health and spiritual care. *Nurs. Stand.* **17**, 33–38.
20. Anandarajah, G. and Hight, E. (2001) Spirituality and medical practice: using the HOPE questions as a practical tool for spiritual assessment. *Am. Fam. Physician* **63**, 81–88.
21. Jonas, W.B. (1998) Alternative medicine — learning from the past, examining the present, advancing to the future. *JAMA* **280(18)**, 1616–1618.
22. Mak, K.Y. and Yip, K.S. (1997) The importance of an effective psychiatric community care service for chronic mental patients in Hong Kong. *Hong Kong J. Ment. Health* **26**, 28–35.
23. Yip, K.S. (1996) A historical review of the community care movement in psychiatric services. *Hong Kong J. Psychiatry* **6(1)**, 38–44.
24. Yip, K.S. (1997) An overview of the development of psychiatric rehabilitation services in Hong Kong. *Hong Kong J. Psychiatry* **26**, 8–27.
25. Ng, H.Y. and Shek, D.T.L. (2001) Religion and therapy: religious conversion and the mental health of chronic heroin-addicted persons. *J. Relig. Health* **40(4)**, 399–410.
26. Hospital Authority. (2001) Position Statement on Holistic Nursing. Hospital Authority, Nursing Section, Hong Kong.
27. Powell, T.J. (1985) Improving the effectiveness of self help. *Soc. Policy* **16(2)**, 22–29.
28. Pargament, K.I. and Brant, C.R. (1998) Religion and coping. In *Handbook of Religion and Mental Health*. Koenig, H.G., Ed. Academic Press, San Diego, CA.
29. Norton, S., Wandersman, A., and Goldman, C.R. (1993) Perceived costs and benefits of membership in a self-help group: comparisons of members and non-members of the Alliance for the Mentally Ill. *Community Ment. Health J.* **29(2)**, 143–160.
30. Goldberg, D.P. (1972) *The Detection of Psychiatric Illness by Questionnaire*. Oxford University Press, Oxford.
31. Chan, D.W. (1985) The Chinese version of the General Health Questionnaire: does language make a difference? *Psychol. Med.* **15**, 147–155.
32. Shek, D.T.L. (1987) Reliability and factorial structure of the Chinese version of the General Health Questionnaire. *J. Clin. Psychol.* **43**, 683–691.
33. Shek, D.T.L. (1989) Validity of the Chinese version of the General Health Questionnaire. *J. Clin. Psychol.* **45(6**), 890–897.
34. Shek, D.T.L. (1993) The factor structure of the Chinese version of the General Health Questionnaire (GHQ-30): a confirmatory factor analysis. *J. Clin. Psychol.* **49(5)**, 678–684.
35. Rosenberg, M. (1965) *Society and the Adolescent Self-Image*. Princeton University Press, Princeton, NJ.
36. Shek, D.T.L. (1992) "Actual-ideal" discrepancies in the representation of self and significant-others and psychological well-being in Chinese adolescents. *Int. J. Psychol.* **27(3&4)**, 229.
37. Sarason, I.G., Sarason, B.R., Shearin, E.N., and Pierce, G.R. (1987) A brief measure of social support: practical and theoretical implications. *J. Soc. Pers. Relatsh.* **4**, 497–510.
38. Chang, A.M. (1999) Psychosocial Nursing Intervention to Promote Self-Esteem and Functional Independence Following Stroke [PhD Thesis]. The Chinese University of Hong Kong.
39. Crumbaugh, J.C. (1968) Cross-validation of purpose in life test based on Frankl's concepts. *J. Individ. Psychol.* **24**, 74–81.
40. Shek, D.T.L. (1988) Reliability and factorial structure of the Chinese version of the Purpose in Life Questionnaire. *J. Clin. Psychol.* **44(3)**, 384–392.
41. Shek, D.T.L. (1992) Meaning in life and psychological well-being: an empirical study using the Chinese version of the purpose in life questionnaire. *J. Genet. Psychol.* **153(2)**, 185–200.
42. Beck, A.T., Weissman, A., Lester, D., and Trexler, L. (1974) The measurement of pessimism: the Hopelessness Scale. *J. Consult. Clin. Psychol.* **42(6)**, 861–865.
43. Shek, D.T.L. (1993) Measurement of pessimism in Chinese adolescents: the Chinese Hopelessness Scale. *Soc. Behav. Pers.* **21(2)**, 107–120.
44. Leung, K.F., Tay, M., Cheng, S.S.W., and Lin, F. (1997). Hong Kong Chinese Version World Health Organization Quality of Life Measure — Abbreviated Version. Hong Kong Hospital Authority.
45. Trojan, A. (1989) Benefits of self-help groups: a survey of 232 members from 65 disease-related groups. *Soc. Sci. Med.* **29(2)**, 225–232.
46. Humphreys, K. and Noke, J.M. (1997) The influence of post treatment mutual help group participation on the friendship networks of substance abuse patients. *Am. J. Community Psychol.* **25(1)**, 1–16.
47. Luk, L.A. (2004) The Changes of Ex-Mental Patients Attending a Psychiatric Rehabilitation Program with Holistic Care [Unpublished PhD dissertation]. The Chinese University of Hong Kong.
48. Shek, D.T.L. (1988) Behavioral intentions to discharged chronic mental patients in a sample of secondary school students. *Zhonghua xin li xue kan* **30(2)**, 79-88.
49. Shek, D.T.L. and Cheung, C.K. (1990) Occupational trust in discharged chronic mental patients in a sample of Chinese secondary school students. *J. Community Psychol.* **18**, 120–129.
50. Larson, D.B., Sherrill, K.A., and Lyons, J.S. (1992) Association between dimensions of religious commitment and mental health reported in the American Journal of Psychiatry and the Archives of General Psychiatry: 1978 through 1989. *Am. J. Psychiatry* **149**, 557–559.
51. Pullen, L., Modrcin-Talbott, M.A., West, W.R., and Muenchen, R. (1999) Spiritual high vs. high on spirits: is religiosity related to adolescent alcohol and drug abuse? *J. Psychiatr. Ment. Health Nurs.* **6**, 3–8.
52. Powell, L.H., Shahabi, L., and Thoresen, C.E. (2003) Religion and spirituality: linkages to physical health. *Am. Psychol.* **58**, 36–52.
53. Riessman, F. (1987) Foreward. In *Self-Help Organization and Professional Practice*. Powell, T.J., Ed. National Association of Social Workers, Silver Spring, MD. pp. ix–x.
54. Pargament, K.I. and Park, C.L. (1995) Merely a defense? The variety of religious means and ends. *J. Soc. Issues* **51**, 13–32.
55. Pargament, K.I. (1997) *The Psychology of Religion and Coping: Theory, Research, Practice*. Guilford, New York.
56. Sullivan, W.P. (1993) It helps me to be a whole person: the role of spirituality among the mentally challenged. *Psychosoc. Rehabil. J.* **16**, 125–134.
57. Chan, C., Wong, D., Ho, P., Ip, F., and Tong, H. (1992) Report of a Survey of the Members of Self-Help Groups for Persons with Chronic Illness in Hong Kong. University of Hong Kong, Department of Social Work & Social Administration.
58. Mok, B.H. (2001) The effectiveness of self-help groups in a Chinese context. *Soc. Work Groups* **24(2)**, 69–89.

## BIOSKETCHES

**Andrew L. Luk, RN, MN, MHA, PhD**, is associate professor of the Kiang Wu Nursing College of Macau. He has a strong commitment to the use of holistic principles in nursing.

**Daniel T.L. Shek, PhD, FHKPs.S, BBS, JP**, is professor at the Department of Social Work, The Chinese University of Hong Kong. He is on the editorial board of several scientific journals and has published extensively in international journals on social work, scale construction, mental health, well being, quality of life, adolescence, disadvantaged families, and drug abuse. E-mail: DANIELSHEK@CUHK.EDU.HK. Website: http://www.cuhk.edu.hk/swk
